# Towards equity in health: for an agenda for research and development and local production driven by the multiple needs of the Brazilian Unified National Health System

**DOI:** 10.1590/0102-311XEN073623

**Published:** 2023-09-18

**Authors:** Mady Malheiros Barbeitas, Alila Brossard Antonielli, Gabriela Costa Chaves, Koichi Kameda

**Affiliations:** 1 Casa de Oswaldo Cruz, Fundação Oswaldo Cruz, Rio de Janeiro, Brasil.; 2 French National Centre for Scientific Research, Paris, France.; 3 Humboldt University of Berlin, Berlin, Germany.; 4 Escola Nacional de Saúde Pública Sergio Arouca, Fundação Oswaldo Cruz, Rio de Janeiro, Brasil.; 5 Núcleo de Estudos e Pesquisas Interdisciplinares sobre Emergências em Saúde Pública, Fundação Oswaldo Cruz, Rio de Janeiro, Brasil.; 6 Population and Development Center, Paris Cité University, Paris, France.

## Introduction

In her inaugural speech on January 2, 2023, the new Brazilian Minister of Health, Nísia Trindade Lima, pointed to local production and the strengthening of the Health Economic-Industrial Complex (CEIS, acronym in Portuguese) as one of her priorities, in addition to resuming the coordination role of the Ministry of Health in defining the needs and stimulating research and the production of strategic inputs for the Brazilian Unified National Health System (SUS, acronym in Portuguese). A public commitment of such magnitude invites us to look to the recent past in order to derive lessons for the present.

During the SARS-CoV-2 pandemic, Brazilian science and technology institutions played a key role in combating it by, for example, promoting rapid access to COVID-19 vaccines even in the face of the Federal Government’s lack of coordination and science denialism. At the same time, the pandemic has exposed Brazil’s great vulnerability: its dependency on importing components for the production of vaccines, drugs, diagnostic tests, equipment and even other less advanced technology items, but equally important in the health context. According to the new Minister, the government’s goal is to locally produce at least 70% of the inputs related to health techologies for the SUS.

One pressing question refers to the scope of what is considered 70% of the inputs: is it related to the volume of units procured, to public spending on health technologies acquired by the SUS, or to a list of the National Essential Medicines List (RENAME, acronym in Portuguese)?

Considering the importance of producing both final products and active pharmaceutical ingredients (API), it is also important to question which segments will be targeted by this effort.

These issues become crucial as access barriers are not limited to availability and encompass other challenges that reflect the dynamics of the current pharmaceutical sector. We address these access barriers from concrete cases involving different health technologies (medicines, diagnosis and vaccines), explaining some elements of this intricate architecture. Our reflection does not address medical equipment.

## Patent, price and pharmaceutical production

Access barriers to newer medicines also include high prices due to the monopoly resulting from patent protection. Since the 1990s, the SUS has felt the weight of the incorporation of HIV medicines (ART) under patent protection. As a result, a series of government initiatives were undertaken to increase the negotiation power of price in government procurements. This included estimates of production cost and addressing patent barriers, such as threat and use of the compulsory license to import and subsequent national production of the efavirenz, both API and the final product ^1^. This lesson cannot be forgotten, since the incorporation of high-priced medicines under patent monopoly only increased over the years.

If the 70% to be produced locally is based on the percentage of public spending on drugs under monopoly compared to the total public expenditure on medicines, we may likely reach only a small list of products.

Local production will need to be accompanied by use of the safeguards set out in the industrial property legislation that favor technological development, such as experimental use, Bolar exemption, patent oppositions and compulsory licensing.

Our technological dependence is reflected not only in the deficit trade balance in the pharmaceutical sector, but also in the vulnerabilities faced by the SUS due to the pharmaceutical practices employed by companies to ensure medicines exclusivity in public procurement, as documented in the literature. The case of ecolizumab is illustrative, since the delay to apply for market authorization (registration) in the country had repercussions on price regulation by the public sector and consequent procurements at high prices, in the context of judicialization ^2^.

Business practices that produce vulnerabilities to pharmaceutical services in the SUS must be on the radar of the selection of candidate items for local production investments and such initiatives should be associated with addressing the generated monopoly.

## Partnerships for productive development

The partnerships for productive development (PDP, acronym in Portuguese), initiated in 2009, relied on a model of technology transfer from the private sector to the public sector (Official Pharmaceutical Laboratories - LFO, acronym in Portuguese) based on a list of strategic products acquired by the SUS. It contemplated the need to include a partner capable of producing API and other inputs in the technology development chain to strengthen this industrial segment in the national territory. The main incentive of the arrangement centered on the guarantee of public market exclusivity to the partners involved during the technology transfer period which, with the evolution of legislation, could reach up to 10 years.

Despite the centrality given to LFOs, PDP implementation has raised many questions regarding the selection of technologies, the limitations of technology capacity building, and the ability to address access barriers to current and future technologies, requiring improvement and other investments to ensure technological absorption and accumulation ^3^ ^,^ ^4^. However, these efforts enabled the Institute of Technology in Immunobiologicals (Bio-Manguinhos), Oswaldo Cruz Foundation (Fiocruz), and the Butantã Institute, during the COVID-19 crisis, to enter into partnerships to ensure the timely supply and national production of two vaccine platforms.

## Investing in neglected tropical diseases

Another aspect of access to be urgently addressed by the new Ministry of Health concerns unmet health needs under the current pharmaceutical research and development (R&D) model. One example would be to support the development and production of medicines, vaccines and diagnostic tests for neglected tropical diseases (NTDs). These diseases affect not only Brazil, but also other countries of the Global South, and are mostly unprofitable.

Brazil had been on an upward investment curve for these diseases. Between 2007 and 2018, the country invested USD 66 million in R&D projects for these diseases ^5^. Unprecedented calls for proposals were created by the Ministry of Health and numerous projects were contracted by the Brazilian Social and Economic Development Bank (BNDES, acronym in Portuguese), taking advantage of research groups and a history of collaboration in this area. As of 2017, these investments declined, but are currently expected to recover, especially after the Federal Government’s commitment to eliminate some NTDs. However, it is paramount to assess what the real legacies of previous calls and funding programs have been in terms of technological learning and a greater understanding of innovation (or its barriers) in the field of NTDs.

Although the figures indicate a trend towards greater prioritization of neglected diseases from 2007 to 2017, they are not sufficient to understand how the innovation system is articulated and operationalized when it comes to introducing a new technology into the market (or in the SUS). In this case, qualitative studies would be important to reveal the learnings and operational barriers when there is R&D funding for NTDs. One example is the fixed-dose combination of artesunate and mefloquine (ASMQ). Its development contributed to the learning and training of Brazilian science and technology institutions, but the product was not fully incorporated into the SUS.

Regarding operational support in the pharmaceutical development chain, Fiocruz has created a clinical research platform, an organization that acts as a public “contract research organization” (CRO), supporting and funding clinical trials for neglected diseases ^6^.

## Vaccines and diagnostic tests

Brazil has capacity but also gaps in the production and development of vaccines and diagnostic tests. Public investment in the national production of these technologies has occurred through partnerships for technology transfer, even before the PDPs. This strategy follows a pragmatic logic to accelerate product availability in the SUS. Moreover, it allows access to other key elements in production of biologicals, such as know-how. This has enabled the public sector to add to its portfolio a set of technologies and platforms that not only meet existing government demands, but can be mobilized for future health emergencies. One example is the nucleic acid test (NAT), whose platform, developed by a national public consortium (Bio-Manguinhos, Federal University of Rio de Janeiro, Molecular Biology Institute of Paraná and Hemobrás) initially for use in blood centers was later mobilized to develop a diagnostic test for arboviruses and COVID-19 ^7^.

While these strategies have favored access to platforms and products, they do not address the production of a number of inputs needed to obtain the final product. In the case of a PCR test, the country lacks the production of primers, probes and pipette tips. This focus on the final product also overlooks other vulnerabilities. One is the need for infrastructure to develop and produce new vaccines and tests ^8^. Some initiatives have sought to address these gaps, such as Bio-Manguinhos’ Henrique Penna Center, which has a pilot plant; Butantã’s Multipurpose Vaccine Production Center, which provides for a security level 3 area; and the future National Vaccine Center of the Brazilian Ministry of Science Technology and Innovation.

Investing in urgent new technological solutions should not prevent public institutions from exercising its primary vocation of meeting the demands that are not of interest to the private sector. One such case is the Montenegro test for cutaneous leishmaniasis. Despite being a priority for the SUS and other countries in the region and the existence of LFO technological capacity, this test is unavailable in Brazil due to a lack of prioritization in the political agenda ^9^.

## Focus on a regional strategy

The COVID-19 pandemic has demonstrated the fragility caused by the specialization of medicines and API production in China and India. These countries halted production and limited exports, prioritizing supply to meet domestic needs.

International cooperation to foster and improve the quality of local production is strategic to address these weaknesses. However, technology transfer initiatives between countries of the Global South require a strong alignment of priorities at the regional and international level, associated with a high-level sustained articulation in international forums and organizations, as well as long-term financial commitment and lengthy projects to develop API production capabilities and increase quality ^10^.

The joint declaration of the Ministries of Health of Argentina and Brazil on January 23, 2023, as well as the meeting of the Mercosur ad hoc committee to promote the expansion of regional production capacity in health technologies, highlights the importance of cooperation between neighbors for the medicines and vaccines R&D. Both initiatives of pooling of efforts and complementarities must be accompanied by concrete elements such as planning and perennial financing lines to get off the ground.

## Final considerations

SUS’ principles of universality, equity and integrality should guide an innovation agenda driven by national and regional health needs that, in many cases, do not present commercial interest but persist in their health essentiality.

The examples discussed in this article illustrate the importance of an industrial and innovation agenda to fully meet the needs of SUS, from new and high-cost technologies, old technologies and at risk of shortages, R&D infrastructure for timely responses to future health emergencies, to technological gaps for NTDs. Announcements that point to South-South cooperation in the region are certainly promising, with a view to mutualizing knowledge, collaborating for common regional needs and - why not - betting on a interdependence dynamic between countries to build a regional health sovereignty that ensures a shared agenda of production and access to health technologies.
